# Therapeutic Potential of Water‐Based Anatolian Propolis Extract Against CAF Diet‐Induced Obesity and Its Metabolic Complications

**DOI:** 10.1002/fsn3.72160

**Published:** 2026-07-28

**Authors:** Mehmet Kemal, Elif Şahin, Ali Kulaber, Sevil Kör, Ahmet Alver, Engin Yenilmez, Sevgi Kolaylı

**Affiliations:** ^1^ Department of Nutrition and Dietetics, Faculty of Health Sciences Karadeniz Technical University Trabzon Turkiye; ^2^ Graduate School of Natural and Applied Science Karadeniz Technical University Trabzon Turkiye; ^3^ Department of Medical Biochemistry, Faculty of Medicine Karadeniz Technical University Trabzon Turkiye; ^4^ Department of Histology and Embryology, Faculty of Medicine Karadeniz Technical University Trabzon Turkiye; ^5^ Department of Chemistry, Faculty of Sciences Karadeniz Technical University Trabzon Turkiye

**Keywords:** anti‐inflammation, antioxidant, C57BL/6, obesity, propolis

## Abstract

This study investigates the anti‐obesity effects of water‐based Anatolian propolis extract in mice with a cafeteria diet‐induced obesity model. The phenolic composition, antioxidant capacity, and α‐amylase/α‐glucosidase inhibitory effects of the WBAP extract were characterized in vitro. For the in vivo study, 42 male C57BL/6 mice were divided into a control group (*n* = 6) and a study group (*n* = 36) fed a CAF diet for 12 weeks. Obese mice were then treated for 4 weeks with WBAP or Orlistat via intraperitoneal injection. At the end of the 28‐day treatment period, serum and tissues were collected. Serum levels of insulin, leptin, adiponectin, TNF‐α, IL‐6, UCP‐1, and various biochemical parameters were analyzed. Liver and adipose tissues were subjected to histopathological analysis. Compared to the untreated CAF group, the WBAP‐treated group exhibited significant reductions in body weight, serum triglycerides, AST, ALT, HOMA‐IR, leptin, and TNF‐α levels. Furthermore, there was a significant increase in adiponectin levels in the WBAP‐treated group. Water‐based Anatolian propolis (WBAP) ameliorates key features of diet‐induced obesity, including dyslipidemia, liver damage, inflammation, and hormonal dysregulation. These results suggest that WBAP is a promising natural and alternative agent for the prevention and treatment of obesity and its related metabolic complications.

## Graphical abstract

Water‐based Anatolian propolis extract attenuates CAF diet‐induced obesity‐related metabolic disturbances by improving dyslipidemia, hepatic injury, adipokine imbalance, and inflammatory status in C57BL/6 mice.

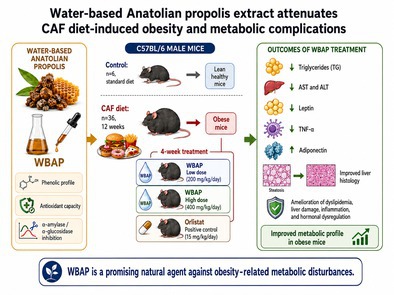

## 1 Introduction

Obesity has become a global health problem over the past few years. This disorder is marked by abnormal or excessive fat deposits. The World Health Organization notes that the incidence of obesity has more than doubled since 1975, with 650 million people older than the age of 18 defined as obese in 2016. Alarmingly, it was reported that almost 40 million children under the age of 5 were overweight and obese in 2020 (World Health Organization 2024). This dramatic increase is largely driven by modern lifestyle changes, notably in eating habits. Obesity is not only associated with a number of serious medical complications but also increases the risk of diseases such as coronary heart disease, some cancers, type II diabetes, and hypertension (Bray et al. 2017; Chang and Kim 2019). Therefore, developing effective strategies for the prevention and therapy of obesity is crucial for public health.

The main goal of obesity therapy is to lower the risks of health problems and death that come with being overweight. This is done along with efforts to improve quality of life, which includes changing habits that lead to obesity and making sure that people eat a balanced and healthy diet. Traditional methods used in the treatment of obesity can be grouped under the titles of medical nutrition therapy, exercise, pharmacological treatment, psychological approaches, and bariatric surgery (Berberoğlu and Hocaoglu 2021). Pharmacological interventions approved by the FDA for prolonged administration use are bupropion/naltrexone‐SR, orlistat, phentermine/topiramate‐ER, and liraglutide. These drugs generally reduce appetite and increase satiety (Calderon et al. 2022). Nevertheless, their long‐term use is frequently constrained by significant side effects, including fat‐soluble vitamin deficiencies, gastrointestinal distress, insomnia, and dizziness (Erdoğan Erden et al. 2022). Due to these limitations, there has been a recent and significant shift in interest toward natural supplements as a complementary approach for weight management. This trend is reflected in a rapidly growing global market for weight‐loss supplements, with annual sales reaching an estimated 30 billion dollars (Koncz et al. 2021).

Propolis is a mixture of resins and bee products gathered from the branches, leaves, wood, and blossoms of some plants by *Apis mellifera* L. and other species. Propolis exhibits anti‐inflammatory, anticarcinogenic, antibacterial, antioxidative, and antifungal properties as well as various medical advantages. Therefore, the application of propolis in health supplements and bio‐cosmetic products is rapidly increasing (Mountford‐McAuley et al. 2023).

Türkiye is known for its location at the crossroads of the Euro‐Siberian, Irano‐Turanian, and Mediterranean regions. This unique geographical location makes Türkiye home to a rich flora with more than 11,000 plant species. This plant diversity in our country explains the differences in the chemical structure of propolis produced (Guzelmeric et al. 2021). Numerous investigations into the composition of Anatolian propolis have demonstrated that Anatolian propolis is abundant in phenolic acids such as *p*‐OH benzoic acid, *p*‐coumaric acid, *t*‐cinnamic acid, ferulic, and caffeic, as well as derivatives such as epicatechin, chrysin, Caffeic acid phenyl ester (CAPE), pinosembrin, luteolin, and flavonoids (El Adaouia Taleb et al. 2020; Guler et al. 2021; Kurek‐Górecka et al. 2022).

The consumption of propolis has grown significantly due to its availability in the trades. Raw propolis collected from beehives is extracted with solvents of different polarities and then consumed as various commercial forms, from direct extracts to capsules and lozenges. Although 70% ethanol is scientifically considered the optimal solvent for maximizing the extraction of bioactive compounds (Kara et al. 2022b), consumer preferences have driven the development of effective alternatives. In response to a growing demand for non‐alcoholic options, particularly for children, preparations such as water‐based extracts, vegetable oil extracts, and polyalcohol‐based extracts have become highly preferred supplements on the market (Yıldız 2020).

The primary objective of this investigation was to examine the anti‐obesity potential of a water‐based Anatolian propolis (WBAP) extract in obese C57BL/6 mice. The findings are intended to not only advance the understanding of obesity pathophysiology but also to support the development of novel therapeutic agents.

## 2 Materials and Methods

### 2.1 Experimental Animals and Study Design

This study utilized 42 male C57BL/6 mice, aged 5–6 weeks and weighing 16–18 g, sourced from the Surgical Research Center at Karadeniz Technical University Faculty of Medicine. A 12‐h dark and 12‐h light environment was maintained for the mice. Food and water were given *ad libitum*.

All animal experiments were approved by the Karadeniz Technical University, Faculty of Medicine, Animal Experiments Local Ethics Committee, Türkiye, under protocol number 2021/3. All procedures were conducted in accordance with institutional and national guidelines for the care and use of laboratory animals.

Mice were accepted after weighing using a precision balance (Venezia Electronic Compact Scale SF‐400A, China) and placed in cages (RAIR IsoSystem, Seaford, DE, USA).

Mice were randomly divided into two groups: a control group (*n* = 6) and an experimental group (*n* = 36). Mice in the control group were fed a rodent diet (RD) produced by Optima Besin Maddeleri (Kırklareli, Türkiye) for 16 weeks. Mice in the experimental group were fed a cafeteria diet (CAF) prepared by Arden Research and Experiment (Ankara), adhering to the OpenSource Diets that were established by Research Diets Inc. The CAF diet is formulated from highly palatable foods such as fruitcakes, chocolate wafers, biscuits, cocoa cream cakes, potato chips, cornstarch, whey protein, and skimmed milk powder. In addition, the cafeteria diet was supplemented with a standard rodent diet due to its low levels of vitamins, minerals, and trace elements. The resulting cafeteria diet had an energy density of 5.004 kcal/g. The energy and macronutrient contents of RD and CAF are shown in Tables S1 and S2.

The body weights of the mice were measured weekly between 16:00 and 18:00 h for 16 weeks using a scale (Venezia Electronic Compact Scale SF‐400A, China) with 0.1 g precision. After the experiment group was fed the CAF for 12 weeks, the mice were weighed. Mice with a weight gain of 8% to 20% compared to their initial weight were considered obese (Gün 2020; Thibault 2013).

**FIGURE 1:**
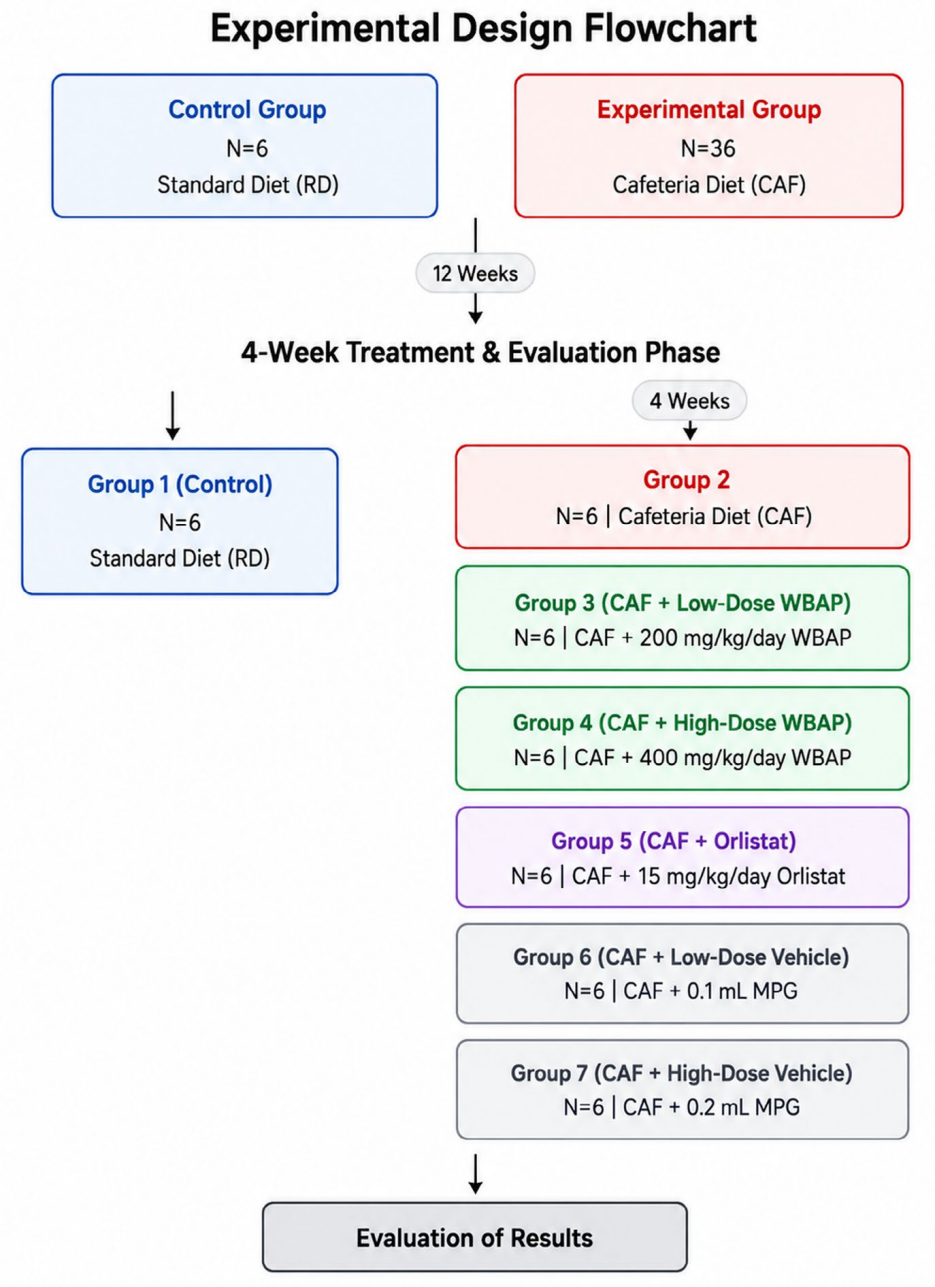
Experimental plan.

Mice considered obese in the experiment group were randomly assigned to one of 6 subgroups (*N* = 6 per subgroup). WBAP (low and high doses), orlistat (ORL), and monopropylene glycol (low and high doses, serving as vehicle) were administered to the respective subgroups by oral gavage for 4 weeks, while continuing the CAF diet. The final experimental groups were as follows: (1) Control group (*N* = 6): mice fed with the RD; (2) Cafeteria diet group (CAF; *N* = 6): mice fed with the CAF; (3) Cafeteria diet and low‐dose WBAP group (CAF + LD‐WBAP; *N* = 6): mice fed with the CAF and 200 mg/kg/day WBAP; (4) Cafeteria diet and high‐dose WBAP group (CAF + HD‐WBAP; *N* = 6): mice fed with CAF and 400 mg/kg/day WBAP; (5) Cafeteria diet and orlistat group (CAF + ORL; *N* = 6): mice fed with CAF and 15 mg/kg/day ORL; (6) Cafeteria diet and low‐dose vehicle group (CAF + LD‐Vehicle; *N* = 6): mice fed with the CAF and 0.1 mL MPG; (7) Cafeteria diet and high‐dose vehicle group (CAF + HD‐Vehicle; *N* = 6): mice fed with the CAF and 0.2 mL MPG. Orlistat was administered at a dose of 15 mg/kg/day, based on previous experimental studies using orlistat in diet‐induced obesity models (Alshagga et al. 2020; Park et al. 2019). All stages of the study process are given in Figure 1.

At the end of the 16‐week experimental period, the mice were fasted for 12 h. The mice were then weighed and sacrificed by the decapitation method. Blood samples were collected in a gel separation tube, and the serums were extracted and preserved at −80°C until the day of analysis. Epididymal adipose tissue (EAT) and liver tissue samples from the mice were removed and weighed. They were subsequently reserved for histopathological examinations. The adiposity index (%) was calculated as follows: (weight of epididymal adipose tissue (EAT) (g) / total body weight (g)) * 100, using the weighed EAT values.

### 2.2 Analysis of Serum Biochemical Parameters

Amylase was measured in serum samples as an indicator of pancreatic enzyme activity, while ALT and AST were evaluated as markers of hepatic injury. In addition, serum glucose, triglyceride, and cholesterol levels were determined. All these parameters were measured using the Beckman Coulter Analyzer AU 5800 autoanalyzer at the KTU Farabi Hospital Medical Biochemistry Laboratory.

### 2.3 Analyses Performed With ELISA Kits

Serum leptin (Cat. No. M0B00B R&D Systems, USA), insulin (Cat. No. E‐EL‐M1382 Elabscience, USA), adiponectin (Cat. No. E‐EL‐M0002 Elabscience, USA), TNF‐α (Cat. No. E‐EL‐M0049 Elabscience, USA), IL‐6 (Cat. No. E‐EL‐M0044 Elabscience, USA), and UCP‐1 (Cat. No. E‐EL‐M0717 Elabscience, USA) levels were measured using commercial mouse‐specific ELISA kits according to the manufacturer's instructions. In addition, the homeostatic model assessment (HOMA) method was applied based on fasting glucose and insulin values to determine insulin resistance in mice, and the HOMA‐IR value was calculated. The calculation was performed using the formula HOMA‐IR = [(insulin (μU/mL) X fasting glucose (mmol/L))/22.5] (Garg et al. 2011).

### 2.4 Histopathological Evaluation of Liver and Adipose Tissue

According to the light microscopic examination, the liver and EAT samples were placed in a 10% formaldehyde solution and fixed for 48 h. The samples were embedded in paraffin blocks, and sections 4–5 μm thick were obtained using an automatic microtome (Leica RM 2255, Tokyo, Japan). The sections were stained with Hematoxylin and Eosin (HandE) on a slide. The histological structures of the tissues were examined using an Olympus BX51 microscope (Olympus, Tokyo, Japan), and photographs were taken using an Olympus DP71 camera (Olympus, Tokyo, Japan) attachment. An experienced histologist evaluated the preparations obtained without knowledge of the working groups. Liver sections were evaluated for vascular congestion, hepatocyte degeneration, sinusoidal dilatation, and congestion in dilated sinusoids, with each feature scored on the following five‐point scale: 1 = Normal/Near Normal, 2 = Mild, 3 = Moderate, 4 = Severe, 5 = Very Severe. The size characteristics of fat cells in adipose tissue were evaluated. 10 images were taken from each experimental group using a 10^X^ objective lens and were used to investigate differences between groups (Sahin et al. 2022).

### 2.5 Water‐Based Anatolian Propolis Extract Sample

The study used water‐based Anatolian propolis extract produced by BEE&YOU SBS Scientific Bio. Solutions Industry and Trade Inc., Istanbul, Türkiye. The propolis samples were stored in a dark place at 4°C in a refrigerator.

### 2.6 Determination of Total Phenolic Content (TPC)

The Folin–Ciocalteu method was used to determine total phenolic content in a sample, using gallic acid as a reference standard and calibrating a calibration graph to measure light absorption (Slinkard and Singleton 1977).

### 2.7 Determination of Total Flavonoid Content (TFC)

The method used to figure out the total flavonoids was the protocol established by (Fukumoto and Mazza 2000). The colorimetric procedure measures the absorbance of a colored product formed when flavonoids complex with aluminum (III), using quercetin as the standard compound and reporting total flavonoid content in mg QUE/mL.

### 2.8 Ferric Reducing Antioxidant Power (FRAP)

The Ferric Reducing Antioxidant Power (FRAP) assay measures the antioxidant power of a Ferric‐TPTZ complex at 593 nm. A calibration curve with Trolox as the standard was created, and the extract's antioxidant strength was determined by comparing its absorbance to the Trolox graph (Benzie and Strain 1999).

### 2.9 DPPH Free Radical Scavenging Activity

DPPH• (2,2‐diphenyl‐1‐picrylhydrazil) is a synthetic radical that reaches its maximal absorbance at a wavelength of 517 nm. The SC_50_ value was used to convey the results. The SC_50_ value indicates the sample concentration that reduces the radical amount by half. Different concentration levels are used to determine this value. Therefore, measurements were performed at six different concentration levels in our study. Absorbance values at 517 nm were recorded following the interaction of each sample solution with radicals, and an absorbance graph was created against the sample concentrations. Using the exponential equation obtained from the graph, the SC_50_ values of the samples were calculated in mg/mL (Molyneux 2004).

### 2.10 Determination of Phenolic Compositions With HPLC–PDA

The study utilized a liquid–liquid extraction method to analyze phenolic compounds using RP‐HPLC‐PDA. The solvent was removed, the residue was dissolved, and the extract was extracted with various solvents. The resulting sample was injected into a Shimadzu HPLC system (C18 column (250 mm x 4.6 mm, 5 μm; GL Sciences) and equipped with a PDA detector in the 200–800 nm wavelength range), comparing 25 standard phenolic compounds (Kara et al. 2022a).

### 2.11 α‐Amylase and α‐Glucosidase Inhibitory Activities of WBAP Extract

The dinitrosalicylic acid (DNS) method was used to assess the effectiveness of an α‐amylase inhibitor. The sample was mixed with the enzyme and substrate reaction, then stopped with DNS reagent and measured at 540 nm. The sample concentration that inhibited 50% of α‐amylase was given as the IC_50_ value. Acarbose was used as the standard inhibitor (Baltaş 2021; El Adaouia Taleb et al. 2020).

The sample solution was mixed with α‐glucosidase enzyme, phosphate buffer, and substrate solution, and the reaction was stopped by adding Na_2_CO_3_. The IC_50_ value was determined by measuring absorbance at 400 nm, with acarbose as the reference inhibitor (Baltaş 2021; El Adaouia Taleb et al. 2020).

### 2.12 Statistical Analysis

The SPSS (IBM SPSS Statistics 23) program was used for the statistical analysis of the data used in the study. The normality of numerical values was analyzed using “Skewness and Kurtosis”, “Shapiro–Wilk Test,” “Histogram,” and “Variance” analyses. The results of the statistical analyses of numerical data were presented in table as mean ± standard deviation. For data showing a normal distribution and to determine the difference between groups on the data variables, one‐way ANOVA variance analysis and Duncan's test were used. For data not showing a normal distribution and to determine the difference between groups on the data variables, Kruskal–Wallis variance analysis and the Mann–Whitney U test were used. Differences at the *p* < 0.05 level were determined to be statistically significant.

## 3 Results and Discussion

Obesity is related to many factors, such as metabolic disorders such as type 2 diabetes mellitus (T2DM), hypertension, and coronary heart disease. In addition, the pathogenesis of obesity can be characterized by biological mechanisms such as excessive lipid storage capacity in adipocytes and systemic inflammation (Cardinault et al. 2020). An experimental model of obesity was established in C57BL/6 mice for this study. The C57BL/6 mouse is a preferred inbred strain for creating diet‐induced obesity (DIO) models, largely due to its rapid and robust development of adiposity when challenged with a high‐fat diet. Therefore, the 16‐week duration of this study was selected to provide sufficient data for a comprehensive evaluation of the therapeutic efficacy in the C57BL/6 obesity model (Sackmann‐Sala et al. 2012; Sahin et al. 2022). In this study, a comprehensive experimental design was implemented to evaluate the anti‐obesity potential of WBAP over a 16‐week period, involving an initial 12‐week obesity induction phase followed by a 4‐week treatment phase (Figure 1).

### 3.1 Impact of WBAP on Body Weight and Adiposity Indices

Mice fed the CAF diet demonstrated a significant increase in body weight by the end of week 12 when compared to the control group (*p* < 0.05) (Figure S1). The control group's final body weight, recorded at 24.12 ± 0.98 g, was significantly lower than that of the CAF group (*p* < 0.05) (Table 1). This successful establishment of a diet‐induced obesity (DIO) model in C57BL/6 mice aligns with previous research highlighting this strain's rapid development of adiposity when challenged with energy‐dense diets (Bortolin et al. 2018; Cai et al. 2020). The CAF diet was selected for this study because it accurately mimics the Western‐style diet by including highly processed foods rich in salt, sugar, and fat. Therefore, it serves as an effective framework for evaluating therapeutic interventions (Sahin et al. 2022). Among the treatment groups, the Orlistat (CAF + ORL) treatment resulted in the lowest final body weight (30.05 ± 1.04 g). While this was significantly lower than the CAF + LD‐WBAP group (*p* < 0.05), it did not differ statistically from the high‐dose WBAP (CAF + HD‐WBAP) group (*p* > 0.05). In contrast, neither the WBAP doses nor the vehicle treatments resulted in a significant reduction in final body weight compared to the untreated CAF group (*p* > 0.05) (Table 1). This suggests that while WBAP modulates metabolic parameters, its effect on total body mass over the 4‐week treatment period is less significant than that of pharmacological agents like Orlistat.

**TABLE 1:** Body weights, liver tissue and EAT values, and % adiposity index values of the groups.

	Control	CAF	CAF + LD‐WBAP	CAF + HD‐WBAP	CAF + ORL	CAF + LD‐Vehicle	CAF + HD‐Vehicle
Initial body weight (g)	17.22 ± 14^a^	17.36 ± 0.69^a^	17.25 ± 0.57^a^	17.07 ± 0.46^a^	17.17 ± 0.39^a^	16.97 ± 0.31^a^	16.91 ± 0.20^a^
Final body weight (g)	24.12 ± 0.98^c^	33.70 ± 1.89^a^	34.62 ± 5.89^a^	32.02 ± 3.19^ab^	30.05 ± 1.04^b^	33.25 ± 0.60^ab^	34.25 ± 0.38^a^
EAT weight (g)	0.47 ± 0.08^b^	1.33 ± 0.24^a^	1.67 ± 0.74^a^	1.20 ± 0.44^a^	0.70 ± 0.26^b^	1.22 ± 0.11^a^	1.28 ± 0.11^a^
Liver tissue weight (g)	1.21 ± 0.09^a^	1.26 ± 0.16^a^	1.18 ± 0.32^b^	1.26 ± 0.08^a^	1.00 ± 0.08^b^	1.25 ± 0.13^a^	1.23 ± 0.16^a^
%Adipocyte index	1.93 ± 0.29^b^	3.94 ± 0.54^a^	4.64 ± 1.37^a^	3.69 ± 1.12^a^	2.33 ± 0.85^b^	3.66 ± 0.33^a^	3.74 ± 0.31^a^

*Note:* Different letters (a–c) in the same columns are significantly different at the 5% level (*p* < 0.05). Means ±standard deviations.

The distribution of adipose tissue serves as a critical indicator of metabolic health, particularly the relationship between visceral fat and insulin resistance (Kitamura 2019). In this study, epididymal adipose tissue (EAT) weight in the CAF group increased by approximately 182% compared to the control group (Table 1). Histological examination confirmed that this expansion was primarily driven by adipocyte hypertrophy (Figure S1) rather than hyperplasia (Table 1). Orlistat was identified as the only intervention that significantly reduced EAT weight. It also normalized the adiposity index to levels comparable to those of the control group (*p* < 0.05) (Table 1). In contrast, WBAP‐treated groups maintained EAT weights and adiposity percentages similar to the untreated CAF group (*p* > 0.05). This finding suggests that WBAP did not promote the breakdown or mobilization of pre‐existing adipose stores during the 4‐week treatment period. Rather, its beneficial effects may be mainly associated with improving the metabolic handling of excess caloric intake and attenuating obesity‐related biochemical disturbances, such as dyslipidemia, hepatic injury markers, and adipokine imbalance. Therefore, the primary effects of WBAP appear to be metabolic regulation rather than direct fat mass reduction or rapid body weight loss.

Liver weight analysis revealed the highest values in the CAF + HD‐WBAP group (1.26 ± 0.08 g), with significant differences observed among the experimental groups (*p* < 0.05). The marked abnormalities in hepatocyte organization and the presence of dense microvesicular lipid vacuoles in the CAF group indicate hepatic lipid accumulation and tissue injury associated with prolonged CAF diet consumption (Table 2).

**TABLE 2:** Morphological change scores of liver tissue.

	Control	CAF	CAF + LD‐WBAP	CAF + HD‐WBAP	CAF + ORL	CAF + LD‐Vehicle	CAF + HD‐Vehicle
Lymphocyte infiltration	1.00 ± 0.00^c^	4.33 ± 0.51^a^	2.33 ± 0.51^b^	2.16 ± 0.40^b^	2.33 ± 0.51^b^	2.33 ± 0.51^b^	2.33 ± 0.51^b^
Hypoeosinophilic cell	1.00 ± 0.00^a^	4.33 ± 0.51^a^	3.33 ± 0.51^b^	2.33 ± 0.51^c^	2.33 ± 0.51^c^	2.50 ± 0.54^c^	2.66 ± 0.51^c^
Lipid accumulation	1.16 ± 0.40^d^	5.00 ± 0.00^a^	3.33 ± 0.51^b^	2.16 ± 0.40^c^	2.50 ± 0.54^c^	2.50 ± 0.54^c^	2.33 ± 0.81^c^

*Note:* Different letters (a–d) in the same columns are significantly different at the 5% level (*p* < 0.05). Means ± standard deviations.

**FIGURE 2:**
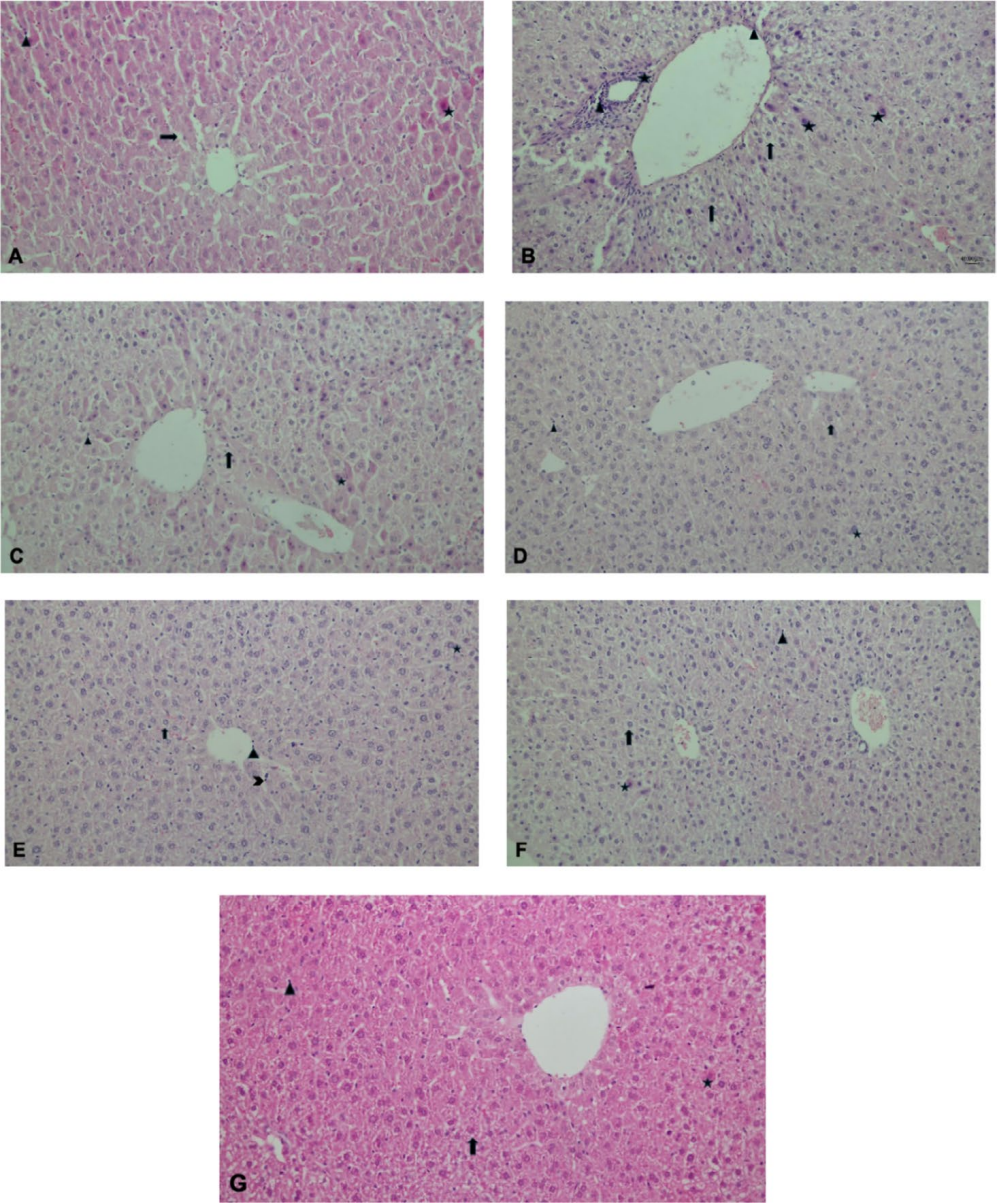
Photomicrograph of the liver tissues. Lymphocyte infiltration (arrowhead), microvesicular lipid vacuoles (arrow), sinusoidal opening (notched arrowhead), hypereosinophilic cell (star) (HandE X 200). (A) Control, (B) CAF, (C) CAF + LD‐WBAP, (D) CAF + HD‐WBAP, (E) CAF + ORL, (F) CAF + LD‐Vehicle, (G) CAF + HD‐Vehicle.

Light microscopic evaluation of liver sections further illustrated the diet‐induced pathological alterations. Compared with the normal hepatic morphology observed in the control group (Figure 2A), the CAF group exhibited disrupted hepatocyte organization and dense microvesicular lipid vacuolization (Figure 2B). Notably, high‐dose WBAP (Figure 2D) and orlistat (Figure 2E) partially attenuated these histological abnormalities, suggesting a shift toward a more preserved hepatic architecture.

The CAF group also exhibited hepatic histopathological alterations, including disrupted hepatocyte organization, lipid vacuolization, and lymphocyte infiltration, which were accompanied by alterations in serum inflammatory mediators such as TNF‐α and IL‐6. However, the present study does not establish a causal relationship between CAF‐induced hepatocellular injury, inflammatory cell infiltration, and systemic inflammatory mediator levels. Therefore, these findings should be interpreted as associated pathological and biochemical changes rather than evidence of a direct mechanistic pathway. Similarly, although WBAP and orlistat improved selected metabolic, biochemical, and histopathological parameters, the current experimental design does not allow definitive conclusions regarding their precise pharmacological mechanisms in this model. Further studies evaluating hepatic inflammatory signaling pathways, oxidative stress markers, immune cell‐specific markers, and molecular regulators of lipid metabolism are required to clarify the mechanisms by which WBAP may modulate CAF diet‐induced metabolic and hepatic disturbances.

### 3.2 Modulation of Serum Biochemical Parameters and Metabolic Profile

The CAF diet induced a state of metabolic dysfunction, characterized by significantly elevated levels of AST, triglycerides (TG), cholesterol, and leptin compared to the control group (*p* < 0.05) (Table 3 and Table S3). These findings validate the C57BL/6 mouse CAF‐model as a representative of human metabolic syndrome. This state is characterized by hyperleptinemia and chronic low‐grade inflammation.

**TABLE 3:** Serum biochemistry parameters.

	Control	CAF	CAF + LD‐WBAP	CAF + HD‐WBAP	CAF + ORL	CAF + LD‐Vehicle	CAF + HD‐Vehicle
Glukoz (mg/dL)	138 ± 21.24^d^	147 ± 26.04^cd^	184.61 ± 27.11^ab^	180.84 ± 24.36^ab^	171 ± 29.36^bc^	171.1 ± 20.56^bc^	204 ± 26.65^a^
ALT (U/L)	29.67 ± 2.33	34.67 ± 10.55	29.98 ± 5.27	25.25 ± 3.76	26.33 ± 3.20	24.58 ± 1.23	23.67 ± 3.88
AST (U/L)	169.33 ± 53.92^b^	243.33 ± 20.96^a^	200.96 ± 58.50^ab^	174.26 ± 45.61^b^	197.33 ± 45.64^ab^	157.37 ± 38.87^b^	189 ± 40.90^ab^
Amylase (U/L)	2295.7 ± 629.55^b^	2697.7 ± 261.19^ab^	3038.1 ± 468.47^a^	2663.6 ± 219.20^ab^	2345.9 ± 238.05^b^	2394.9 ± 273.78^b^	2624 ± 168.52^ab^
Cholesterol (mg/dL)	72.67 ± 14.51	163.67 ± 12.86	169.69 ± 12.05	164.20 ± 14.31	151.56 ± 27.06	151.93 ± 20.51	165.33 ± 14.34
Triglyceride (mg/dL)	74.67 ± 17.09^b^	94.33 ± 13.64^a^	84.51 ± 23.94^ab^	71.92 ± 17.57^b^	69.78 ± 13.65^b^	72.21 ± 8.10^b^	74 ± 11.59^b^
Leptin (pg/mL)	1716.7 ± 425.17	14762.73 ± 7339.36	27548.53 ± 2932.42	13770.85 ± 4621.53	10591.1 ± 5718.02	6677.2 ± 4546.97	7242.41 ± 3358.02
Adiponectin (pg/mL)	4997.07 ± 2659.65	3395.37 ± 1726.08	9553.74 ± 2333.65	9971.41 ± 2374.28	12700.60 ± 16056.41	9498.89 ± 5043.01	11740.28 ± 4999.43
IL‐6 (ng/mL)	2339.22 ± 141.49^d^	2884.37 ± 381.44^c^	2937.8 ± 334.21^bc^	3144.9 ± 162.59^abc^	3241.30 ± 373.31^ab^	3324.39 ± 177.00^a^	3111.62 ± 193.37^abc^
TNF‐α (ng/mL)	1612.19 ± 85.45	1701.36 ± 94.22	1858.12 ± 271.26	1764.63 ± 175.10	1849.01 ± 24.43	1791 ± 43.11	1803.94 ± 41.85
Insülin (pg/mL)	109.49 ± 59.53^a^	137.24 ± 58.77^a^	92.33 ± 31.64^a^	75.31 ± 12.26^a^	77.37 ± 14.03^a^	90.86 ± 19.76^a^	125.96 ± 36.72^a^
UCP‐1 (pg/mL)	9.09 ± 0.77^c^	10.43 ± 1.27^bcd^	9.62 ± 1.18^cd^	11.33 ± 1.39^ab^	10.70 ± 0.46^abc^	10.83 ± 1.16^abc^	11.96 ± 0.98^a^
HOMA‐IR	0.96 ± 0.63^a^	1.21 ± 0.48^ab^	1.08 ± 0.53^ab^	0.84 ± 0.23^a^	0.82 ± 0.24^a^	0.95 ± 0.23^a^	1.56 ± 0.48^b^

*Note:* Different letters (a–d) in the same columns are significantly different at the 5% level (*p* < 0.05). Means ± standard deviations.

#### 3.2.1 Lipid‐Lowering and Hepatoprotective Effects

A result of this study is the high‐dose WBAP (CAF + HD‐WBAP) treatment, which significantly reduced serum TG, AST, and ALT levels compared to the untreated CAF group (*p* < 0.05). This lipid‐lowering efficacy was comparable to the positive control, Orlistat, suggesting that WBAP has potent regulatory effects on obesity‐induced dyslipidemia and hepatotoxicity. These results align with previous studies on Chinese and Croatian propolis, which reported similar reductions in TG and liver enzymes in HFD‐fed mice (Oršolić et al. 2019; Zheng et al. 2020). In addition, while TG levels dropped, serum cholesterol remained stable across WBAP groups (*p* > 0.05), consistent with findings by Koya‐Miyata et al. (Koya‐Miyata et al. 2009). This suggests a specific metabolic pathway for propolis‐mediated TG clearance that does not extend to cholesterol homeostasis.

Although WBAP and orlistat improved some obesity‐associated metabolic parameters, the mechanisms underlying these effects cannot be directly compared based on the current dataset. Orlistat is known to exert its anti‐obesity effect mainly through inhibition of gastrointestinal lipases and reduction of dietary fat absorption. In the present study, fecal triglycerides, fecal total cholesterol, bile acid content, and gastrointestinal lipase activity were not measured. Therefore, it is not possible to determine whether WBAP influenced lipid absorption, fecal lipid excretion, bile acid metabolism, or lipase activity in a manner comparable to orlistat. Accordingly, the effects of WBAP should be interpreted primarily as improvements in serum biochemical parameters, adipokine balance, and liver histopathology rather than as evidence of an orlistat‐like mechanism. Future studies including fecal lipid profiling, bile acid analysis, and lipase activity measurements are required to clarify the precise mechanisms through which WBAP modulates obesity‐related metabolic disturbances.

#### 3.2.2 Glucose Homeostasis and Insulin Sensitivity

Insulin resistance is a key characteristic of obesity‐induced signaling disruption. In this study, while WBAP treatments led to a numerical decrease in serum insulin levels and HOMA‐IR values compared to the CAF group, these changes did not reach statistical significance (*p* > 0.05) (Table 3). However, it is noteworthy that the HOMA‐IR values in the high‐dose WBAP and Orlistat groups remained comparable to those of the control group. This indicates a stabilizing effect on insulin sensitivity. These findings support the role of propolis polyphenols in enhancing insulin sensitivity, consistent with findings reported by (Cai et al. 2020). The ability of WBAP to maintain glucose homeostasis despite the energy‐dense CAF diet underscores its potential as a therapeutic intervention for Type 2 Diabetes prevention.

#### 3.2.3 Adipokine Dysregulation and Inflammation

Adipose tissue acts as a critical endocrine organ; its dysfunction leads to decreased adiponectin and increased leptin (Catta‐Preta et al. 2012). WBAP supplementation effectively corrected this dysregulation by significantly raising serum adiponectin and, at the high‐dose WBAP, lowering leptin levels (*p* < 0.05) (Table 3 and Table S3). This modulation is critical for reversing leptin resistance, a central problem in obesity pathology (Frühbeck et al. 2018).

Regarding systemic inflammation, the high‐dose WBAP was more effective at reducing TNF‐α levels than Orlistat, although this did not reach statistical significance. Conversely, an unexpected dose‐dependent increase in IL‐6 was observed in WBAP groups, with the highest levels recorded in the Orlistat group (Table 3 and Table S3). This divergence from some previous studies suggests that the water‐based extract may regulate inflammatory pathways differently than ethanol‐based extracts, an observation that warrants further molecular investigation (Kitamura 2019).

#### 3.2.4 Thermogenesis and “Browning” Potential (UCP‐1)

The induction of beige adipocytes within white adipose tissue, known as browning, has emerged as a potential target for obesity management. Previous studies have suggested that propolis‐derived compounds, such as Artepillin C found in Brazilian propolis, may stimulate UCP‐1 expression and thermogenic activity (Tsuda and Kumazawa 2021). In the present study, WBAP showed a complex, non‐linear dose–response pattern for serum UCP‐1 levels. Although low‐dose WBAP significantly altered UCP‐1 levels, this effect was not accompanied by a significant reduction in body weight. This discrepancy may be explained by several factors. First, changes in UCP‐1‐related thermogenic signaling may not be sufficient to induce measurable body weight reduction within a relatively short 4‐week intervention period. Second, WBAP may primarily improve the metabolic handling of excess caloric intake rather than directly promoting the mobilization or breakdown of pre‐existing adipose tissue. Therefore, the observed UCP‐1 response may reflect an early or partial thermogenic adaptation, whereas a longer intervention or stronger activation of adipose tissue browning may be required to produce significant reductions in total body mass. Further studies evaluating UCP‐1 expression directly in adipose tissue, together with energy expenditure and mitochondrial activity, are needed to clarify the thermogenic potential of WBAP.

### 3.3 *In Vitro* Characterization and Phenolic Profile of WBAP

The WBAP extract exhibited a robust polyphenolic profile, with a total phenolic content (TPC) of 44 ± 3.28 mg GAE/mL and a total flavonoid content (TFC) of 24.6 ± 0.53 mg QUE/mL. This chemical richness was accompanied by strong antioxidant capacity, as demonstrated by a FRAP value of 1192.571 ± 175.94 mM Trolox/mL and a DPPH SC_50_ of 0.02 ± 0.004 mg/mL (Table 4). The antioxidant and biological activities of propolis are highly dependent on its botanical origin and the secondary metabolites harvested by honeybees from local flora, such as chestnut, pine, and oak (Kolaylı et al. 2023). In this regard, Ören et al. (2024) reported that Siirt propolis extracts obtained using different solvent systems showed DPPH radical scavenging activity ranging from 13.86% to 35.72% and ABTS radical scavenging activity ranging from 33.62% to 62.50% at 30 μg/mL. They also found that extracts with higher phenolic content exhibited stronger enzyme inhibitory activity, with the ethanol extract showing the highest α‐glucosidase inhibition, with an IC50 value of 5.72 ± 0.83 μg/mL. Compared with those findings, the present WBAP extract showed marked antioxidant capacity and strong α‐glucosidase inhibitory activity, although its α‐glucosidase inhibition was weaker than that reported for the ethanol‐rich Siirt propolis extract. This difference may be related to the water‐based formulation of WBAP, since ethanol‐containing extracts generally yield higher amounts of lipophilic phenolics and flavonoids. Nevertheless, the α‐glucosidase inhibitory activity observed for WBAP suggests that water‐based Anatolian propolis may still contribute to the modulation of carbohydrate digestion. In our WBAP sample, chrysin was identified as the most abundant bioactive compound among the 11 detected phenolics (Table 4). In addition to its antioxidant capacity, the WBAP extract demonstrated notable inhibitory activity against α‐amylase and α‐glucosidase enzymes (Table 4). While it showed potent inhibitory activity against α‐glucosidase with an IC50 of 30.79 ± 0.03 μg/mL, its effect on α‐amylase was relatively moderate (3725.02 ± 0.00 μg/mL). This selective inhibition pattern may be metabolically relevant because α‐glucosidase plays a key role in the final stage of carbohydrate digestion and postprandial glucose release (El Adaouia Taleb et al. 2020; Ören et al. 2024). More recently, a systematic review and meta‐analysis of randomized controlled trials reported that propolis supplementation significantly improved fasting plasma glucose, 2‐h postprandial glucose, HbA1c, fasting insulin, and HOMA‐IR in patients with Type 2 Diabetes (Karimi et al. 2025). Therefore, the present findings support the view that WBAP may act as a supportive natural agent for improving obesity‐related glucose metabolism; however, its role in Type 2 Diabetes prevention or management should be confirmed by targeted mechanistic studies and clinical trials.

**TABLE 4:** In vitro properties of WBAP.

***Antioxidant properties***	
Total Phenolic Contents (mg GAE/mL)	44 ± 3.28
Total Flavonoid Contents (mg QUE/mL)	24.6 ± 0.53
Total Antioxidant Capacity‐FRAP (mM Trolox/mL)	1192.571 ± 175.94
DPPH SC_50_ (mg/mL)	0.02 ± 0.004
***Inhibition of α‐amylase and α‐glucosidase***	
α‐amylase inhibition (IC_50_‐μg/mL)	3725.02 ± 0.00
Acarbose (IC_50_‐μg/mL)	88.61 ± 003
α‐glucosidase (IC_50_‐μg/mL)	30.79 ± 0.03
Acarbose (IC_50_‐μg/mL)	7.92 ± 0.02
***Phenolic Profiles (μg/ml)***	
Gallic Acid	< LOD
Protocatechuic acid	< LOD
Chlorogenic acid	< LOD
*p*‐OH Benzoic acid	< LOD
Epicatechin	< LOD
Caffeic acid	89.552
Syringic acid	< LOD
*m*‐OH Benzoic acid	< LOD
Rutin	< LOD
Ellagic acid	< LOD
*p*‐Coumaric acid	48.082
Ferulic acid	91.680
Myricetin	< LOD
Resveratrol	< LOD
Daidzein	< LOD
Luteolin	1.074
Quercetin	11.444
*t*‐Cinnamic acid	27.220
Apigenin	3.028
Hesperidin	4.050
Rhamnetin	< LOD
Chrysin	422.534
Pinocembrin	278.582
CAPE	241.550
Curcumin	< LOD

Abbreviation: LOD, Limit of Detection.

## 4 Conclusion

In conclusion, the present investigation highlights the therapeutic potential of WBAP in mitigating the metabolic consequences of cafeteria diet‐induced obesity. The outstanding results demonstrate that WBAP effectively regulates dyslipidemia, hepatic steatosis, and insulin resistance, with high‐dose treatments showing efficacy comparable to the clinical drug Orlistat in reducing serum triglycerides and liver enzymes. By positively modulating the adipokine profile and suppressing systemic inflammation, WBAP serves as a potent natural agent for maintaining whole‐body metabolic homeostasis. Future research activities should focus on elucidating the specific molecular mechanisms and signaling pathways, such as AMPK or PPAR‐γ modulation, through which WBAP polyphenols exert their anti‐obesity effects. Additionally, further clinical trials are warranted to evaluate the bioavailability of these bioactive compounds in humans and to establish standardized dosage regimens for the prevention and management of metabolic syndrome.

## Author Contributions

**Elif Şahin:** formal analysis, data curation. **Sevgi Kolaylı:** methodology, writing – review and editing, project administration, resources, supervision. **Engin Yenilmez:** supervision, methodology. **Mehmet Kemal:** writing – original draft, data curation, formal analysis, conceptualization, methodology. **Sevil Kör:** formal analysis. **Ali Kulaber:** formal analysis, data curation. **Ahmet Alver:** supervision, methodology, investigation, visualization.

## Funding

This work was supported by the Office of Scientific Research Projects of Karadeniz Technical University, Türkiye, under project number FSI‐2021‐9820.

## Ethics Statement

The animal study was reviewed and approved by the Karadeniz Technical University Animal Experiments Local Ethics Committee, Türkiye, with approval number 2021/3. All procedures involving animals were performed in accordance with the relevant institutional and national guidelines for the care and use of laboratory animals.

## Conflicts of Interest

The authors declare no conflicts of interest.

## Supporting information


**Table S1:** Nutrient Composition of Rodent Diet.
**Table S2:** Nutrient Composition of Cafeteria Diet.
**Table S3:** Statistical comparison of ALT, cholesterol, leptin, adiponectin, and TNFα parameters between groups (Kruskal–Wallis test and Mann–Whitney U test results).
**Figure S1:** Weight change in groups during the 16‐week diet period (mean ± standard deviation).
**Figure S2:** A micrograph of lipid tissue. Arrow indicates a typical adipocyte. Triangel indicates vascular congestion (HandE X 200). A: Control, B: CAF.
**Figure S3:** A micrograph of lipid tissue. Arrow indicates a typical adipocyte. Triangel indicates vascular congestion (HandE X 200). A: CAF + LD‐WBAP, B: CAF + HD‐WBAP, C: CAF + ORL.
**Figure S4:** A micrograph of lipid tissue. Arrow indicates a typical adipocyte. Triangel indicates vascular congestion (HandE X 200). A: CAF + LD‐Vehicle, B: CAF + HD‐Vehicle.

## Data Availability

The data that support the findings of this study are available from the corresponding author upon reasonable request.
