# Supplementary material for: Therapeutic Potential of Water‐Based Anatolian Propolis Extract Against CAF Diet‐Induced Obesity and Its Metabolic Complications
Source: Food Sci Nutr. 2026 Jul 28;14(8):e72160. doi: 10.1002/fsn3.72160 (PMC13415984; doi:10.1002/fsn3.72160)
Supplement: Supplementary file 1 — Table S1: Nutrient Composition of Rodent Diet. Table S2: Nutrient Composition of Cafeteria Diet. Table S3: Statistical comparison of ALT, cholesterol, leptin, adiponectin, and TNFα parameters between groups (Kruskal–Wallis test and Mann–Whitney U test results). Figure S1: Weight change in groups during the 16‐week diet period (mean ± standard deviation). Figure S2: A micrograph of lipid tissue. Arrow indicates a typical adipocyte. Triangel indicates vascular congestion (HandE X 200). A: Control, B: CAF. Figure S3: A micrograph of lipid tissue. Arrow indicates a typical adipocyte. Triangel indicates vascular congestion (HandE X 200). A: CAF + LD‐WBAP, B: CAF + HD‐WBAP, C: CAF + ORL. Figure S4: A micrograph of lipid tissue. Arrow indicates a typical adipocyte. Triangel indicates vascular congestion (HandE X 200). A: CAF + LD‐Vehicle, B: CAF + HD‐Vehicle. [file FSN3-14-e72160-s001.docx]

**Supplementary Information**

**Therapeutic Potential of Water-Based Anatolian Propolis Extract Against CAF Diet-Induced Obesity and its Metabolic Complications**

Mehmet Kemal^a,e^, Elif Şahin^b^, Ali Kulaber^c^, Sevil Kör^b^, Ahmet Alver^b^, Engin Yenilmez^c^, Sevgi Kolaylı^d*^

^a^Department of Nutrition and Dietetics, Faculty of Health Sciences, Karadeniz Technical University, Trabzon, 61080, Türkiye

^b^Department of Medical Biochemistry, Faculty of Medicine, Karadeniz Technical University, Trabzon, 61080, Türkiye

^c^Department of Histology and Embryology, Faculty of Medicine, Karadeniz Technical University, Trabzon, 61080, Türkiye

^d^Department of Chemistry, Faculty of Sciences, Karadeniz Technical University, 61080 Trabzon, Türkiye

^e^Graduate School of Natural and Applied Science, Karadeniz Technical University, 61080 Trabzon, Türkiye

Corresponding Author: Prof. Dr. Sevgi Kolaylı Department of Chemistry, Faculty of Sciences, Karadeniz Technical University, 61080, Trabzon, Türkiye

Email: skolayli@ktu.edu.tr

**Table S1.** Nutrient Composition of Rodent Diet

| **Analytical Components** | **%** | **Trace Elements** | **mg/kg** | **Vitamins** | **IU/Kg** |
| --- | --- | --- | --- | --- | --- |
| Crude protein | 24 | 3b202 Iodine | 1.3 | 3a672a Vitamin A | 36000 |
| Crude fiber | 3.2 | 3b302 Cobalt | 0.15 | E67 Vitamin D3 | 6500 |
| Crude fat | 05.09 | E4 Copper | 16 |  |  |
| Ash | 8.2 | 3b502 Manganese | 155 |  |  |
| Lysine | 1.4 | Zinc oxide | 130 |  |  |
| Methionine | 0.6 | E8 Selenium | 0.35 |  |  |
| Calcium | 1.13 |  |  |  |  |
| Phosphorus | 0.86 |  |  |  |  |
| Sodium | 0.28 |  |  |  |  |

*Values are based on the nutritional label on the Optima feed package.

**Table S2.** Nutrient Composition of Cafeteria Diet

| **Ingredients** | **Amount Used (g)** | **Total Carbohydrate (g)** | **Total Protein (g)** | **Total Fat (g)** | **Total Calories (kcal)** |
| --- | --- | --- | --- | --- | --- |
| Rodent Diet | 1000 | 430.2 | 240 | 55 | 3000 |
| Potato chips | 1000 | 605 | 72 | 245 | 4910 |
| Biscuits | 10000 | 7370 | 800 | 1200 | 44000 |
| Chocolate wafers | 1000 | 640 | 48 | 240 | 5000 |
| Cocoa cream cakes | 500 | 273 | 21.5 | 87.5 | 1975 |
| Fruitcakes | 500 | 280 | 21 | 74.5 | 1890 |
| Cellulose | 500 | 0.00 | 0.00 | 0.00 | 0.00 |
| Oil | 4500 | 0.00 | 0.00 | 4500 | 40500 |
| Corn starch | 2000 | 2000 | 0.00 | 0.00 | 8000 |
| Grape molasses | 1000 | 690 | 14 | 2 | 2830 |
| Skimmed milk powder | 1000 | 800 | 200 | 0.00 | 4000 |
| Whey protein powder | 1000 | 950 | 50 | 0.00 | 4000 |
| Total | 24000 | 1438.20 | 1466.50 | 6404 | 120105 |

**Table S3.** Statistical comparison of ALT, cholesterol, leptin, adiponectin, and TNFα parameters between groups (Kruskal-Wallis test and Mann-Whitney U test results)

| **Groups** | **ALT** | **Cholesterol** | **Leptin** | **Adiponectin** | **TNFα** |
| --- | --- | --- | --- | --- | --- |
| **Control - CAF** | 0.36 | 0.004* | 0.004* | 0.262 | 0.037* |
| **Control - CAF+LD-WBAP** | 0.629 | 0.004* | 0.055 | 0.016* | 0.025* |
| **Control - CAF+HD-WBAP** | 0.05 | 0.004* | 0.004* | 0.010* | 0.025* |
| **Control - CAF+ORL** | 0.07 | 0.004* | 0.004* | 0.631 | 0.004* |
| **CAF - CAF+LD-WBAP** | 0.747 | 0.470 | 0.749 | 0.004* | 0.078 |
| **CAF - CAF+HD-WBAP** | 0.023* | 0.872 | 0.936 | 0.006* | 0.521 |
| **CAF - CAF+ORL** | 0.022* | 0.630 | 0.522 | 0.262 | 0.054 |
| **CAF - CAF+LD-Vehicle** | 0.004* | 0.423 | 0.025* | 0.010* | 0.055 |
| **CAF - CAF+HD-Vehicle** | 0.005* | 0.808 | 0.025* | 0.006* | 0.055 |
| **CAF+LD-WBAP - CAF+HD-WBAP** | 0.147 | 0.630 | 0.631 | 0.522 | 0.148 |
| **CAF+LD-WBAP - CAF+ORL** | 0.107 | 0.200 | 0.423 | 0.423 | 0.335 |
| **CAF+LD-WBAP - CAF+LD-Vehicle** | 0.053* | 0.078 | 0.200 | 0.522 | 0.423 |
| **CAF+LD-WBAP - CAF+HD-Vehicle** | 0.037* | 0.688 | 0.150 | 0.631 | 0.336 |
| **CAF+HD-WBAP - CAF+ORL** | 0.511 | 0.572 | 0.337 | 0.522 | 0.100 |
| **CAF+HD-WBAP - CAF+LD-Vehicle** | 0.870 | 0.228 | 0.016* | 0.575 | 0.172 |
| **CAF+HD-WBAP - CAF+HD-Vehicle** | 0.744 | 0.936 | 0.016* | 0.522 | 0.199 |
| **CAF+ORL - CAF+LD-Vehicle** | 0.324 | 0.810 | 0.109 | 0.631 | 0.025* |
| **CAF+ORL - CAF+HD-Vehicle** | 0.288 | 0.631 | 0.337 | 0.423 | 0.065 |
| **CAF+LD-Vehicle - CAF+HD-Vehicle** | 1.00 | 0.37 | 0.749 | 0.337 | 0.631 |

* p<0.05 indicates significance.

**Figures**


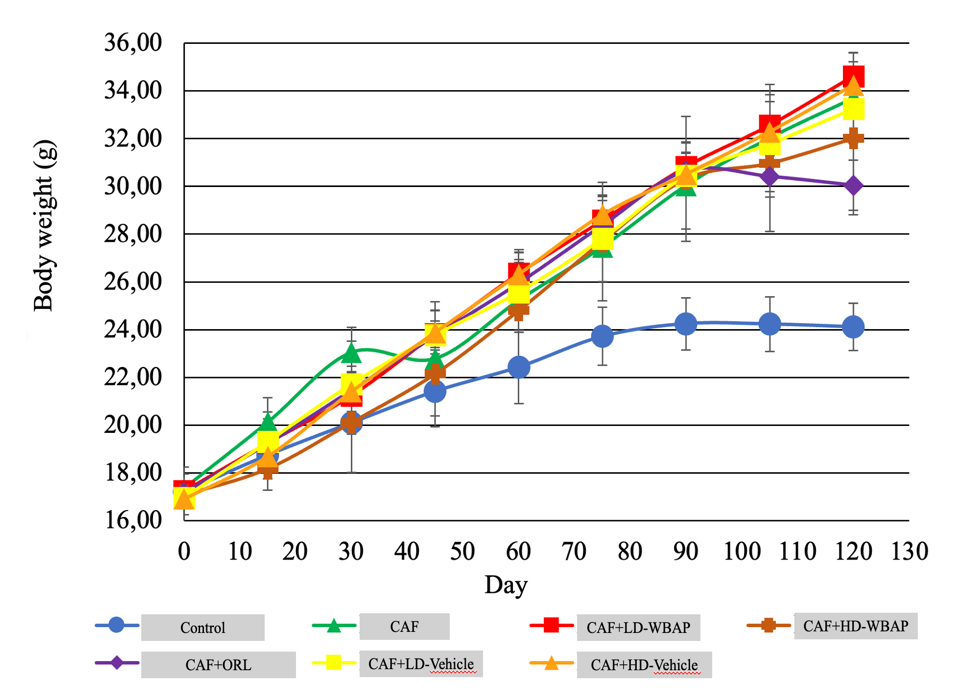


**Fig. S1.** Weight change in groups during the 16-week diet period (mean ± standard deviation)


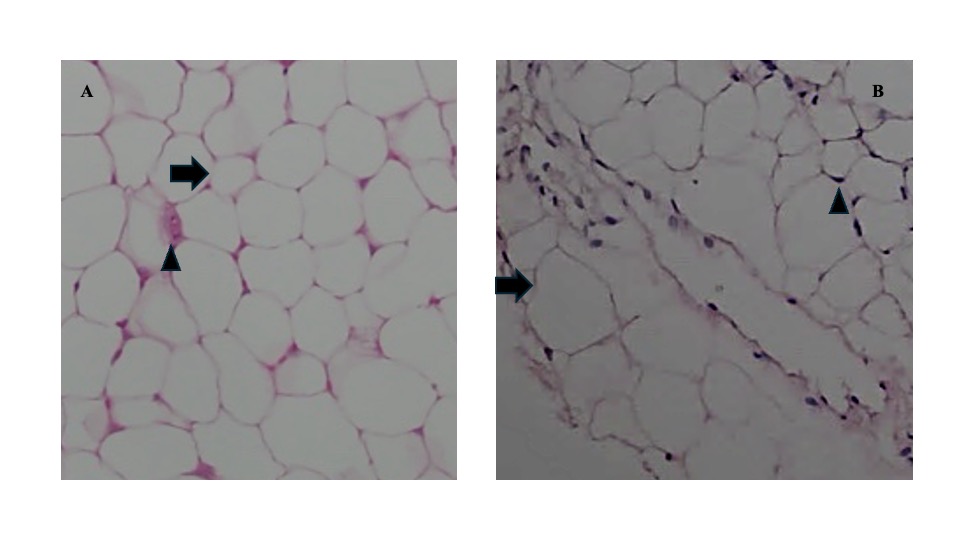


**Fig. S2.** A micrograph of lipid tissue. Arrow indicates a typical adipocyte. Triangel indicates vascular congestion (H&E X 200). A: Control, B: CAF


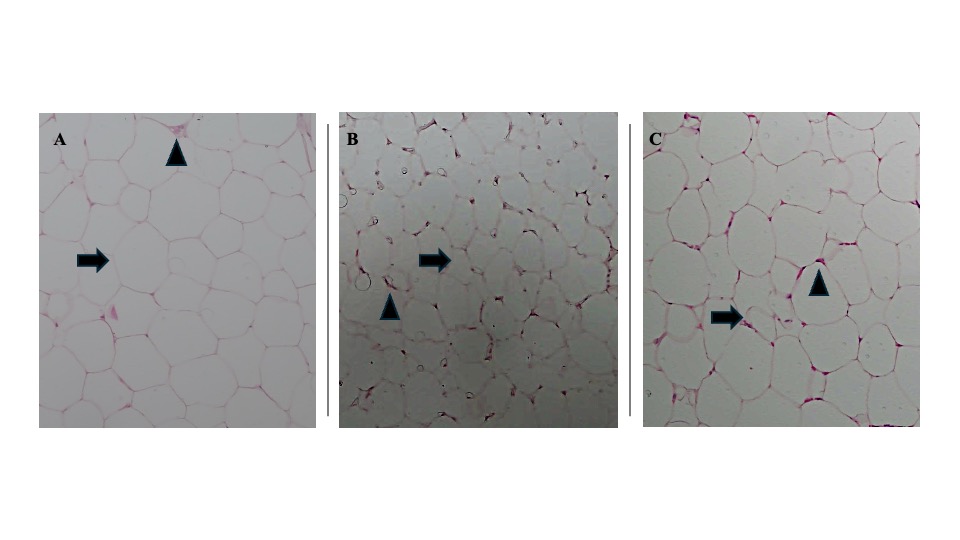


**Fig. S3.** A micrograph of lipid tissue. Arrow indicates a typical adipocyte. Triangel indicates vascular congestion (H&E X 200). A: CAF+LD-WBAP, B: CAF+HD-WBAP, C: CAF+ORL


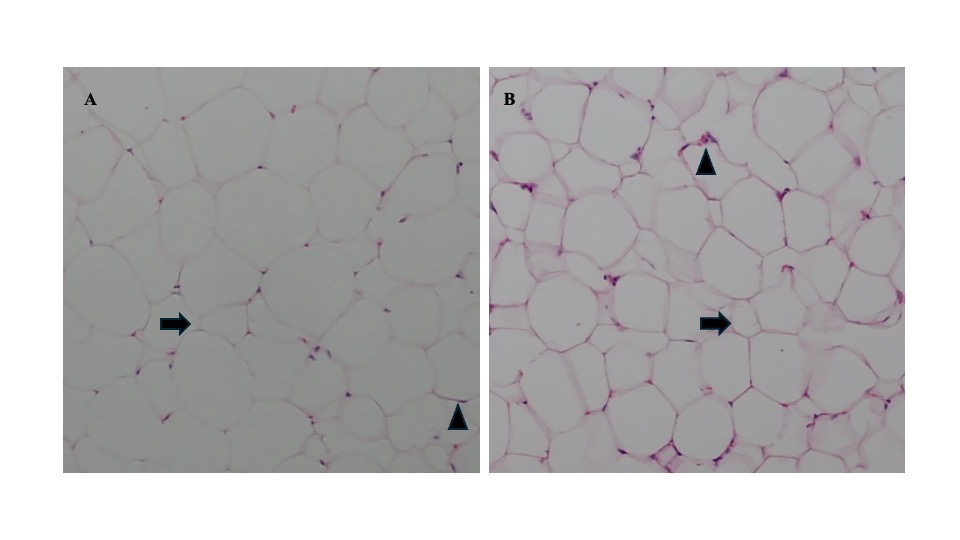


**Fig. S4.** A micrograph of lipid tissue. Arrow indicates a typical adipocyte. Triangel indicates vascular congestion (H&E X 200). A: CAF+LD-Vehicle, B: CAF+HD-Vehicle
